# Advances in Management of Bladder Cancer

**DOI:** 10.3390/jcm11010203

**Published:** 2021-12-30

**Authors:** Marco Racioppi

**Affiliations:** Clinica Urologica, Policlinico “Agostino Gemelli” Foundation, Catholic University of Sacred Hearth, IRCSS, 00168 Rome, Italy; marco.racioppi@policlinicogemelli.it

Bladder cancer (BC) is a complex disease with the following presentations, which are completely different from one another: non-muscle-infiltrating bladder cancer (NMIBC) and muscle-infiltrating bladder cancer (MIBC). This fact gives rise to a wide set of problems concerning different diagnoses, treatments, and follow-up, and currently makes the tumor the most expensive cancer in the world for the healthcare system. All the papers presented in this Special Issue touch on this topic.

What characterizes the tumor is its high prevalence in the population, rather than its incidence, and cigarette smoking is certainly considered the main risk factor. However, other less-considered factors can also play a role today. The long life expectancy increases the number of patients with forms of incomplete urinary emptying, secondary to some types of obstruction; other authors have already highlighted post-voiding urinary residual as a possible risk factor. In their paper, Junghoon et al. show a 2.6-fold increased risk of recurrence in patients with NMIBC when intravesical prostate protrusion is present [1]. I think that this could also be considered as one of the reasons why octogenarians are at high risk for BC or have worse development.

On the other hand, speaking of diagnostic tools, we still do not have real markers for detecting BC. Even now, in the management of NMIBC, we rely on endoscopy. We must say that this has precisely led to technical improvements in the vision-based management of the disease (e.g., photodynamic diagnosis and narrow-band imaging), to allow better management of the neoplasm, but obviously the discomfort for the patient and the high management costs remain. We are looking for new possibilities and new markers, not only for the first diagnosis, but also, and above all, for the follow-up, which have a degree of reliability to replace or at least reduce the number of cystoscopies. Genomic sequencing is helping us and good data come from multiplex assays, which are now under investigation, that detect DNA methylation alterations. However, we must always look to the future. A good urinary bladder cancer marker should have the following characteristics: technically simple, high specificity and high sensitivity, good reliability and reproducibility, and low cost. The paper by Bassi et al. is a pilot study about the possible ability of a simple device, such as the electronic nose, to detect bladder cancer by the evaluation of specific volatile organic compounds in the gas emitted from urine samples [2]. Preliminary data are interesting, but obviously this is the beginning and further studies are required.

The therapy for NMIBC is transurethral resection of the bladder followed by intravesical instillations of chemotherapeutic agents or BCG, while, for MIBC, the best therapies are currently neoadjuvant chemotherapy (NAC) and radical cystectomy (RC). BCG has been one of the biggest advances in the treatment of high-grade NMIBC, but since its introduction, there have been no other dramatically important developments. After BCG failure in high-grade NMIBC, RC is the treatment of choice. Why BCG fails up to 40% of the time is not fully understood. Research on PD-L1 immunosuppressive signaling opens new possibilities not only to understand the possible mechanisms that contribute to BCG failure [3], but also to open new horizons in the therapy of both forms, non-infiltrating and infiltrating, with checkpoint inhibitors. The research into this area is progressing.

RC remains the mainstay of MIBC treatment, along with lymphadenectomy. Should lymphadenectomy be extended or standard? This remains a dilemma. We still do not have a certain answer on the impact of the extension of lymphadenectomy on survival. Leminsky et al. also attempt to find a further correlation between the number of lymph nodes removed, neoadjuvant chemotherapy, and survival [4]. However, the increased use of the currently optimal therapeutic choice, based on NAC and RC, seems to reduce the importance of the number of lymph nodes removed on survival. However, it is necessary to check this finding with larger databases.

Some decades have passed since the pioneering proposal to use the bowel for orthotopic reconstruction of the bladder after cystectomy. Now, the orthotopic neobladder is a frequent choice when considering the possibilities of urinary diversion after cystectomy for BC. Many studies have been conducted on the consequences, including metabolic consequences, that an intestinal diversion can have on the patient (impairment in the absorption of vitamins, metabolic acidosis, bone reabsorption, and alterations in bowel motility), but the impact of the intestinal neobladder on renal function is certainly important. Jihion et al. found no significant differences between the universally known and established non-continent ileal conduit and orthotopic ileal neobladder diversions at a follow-up of 12 months [5]. Urologists must be aware of this because intestinal orthotopic diversions are certainly indicated for younger patients, so it is important to evaluate the impact of the diversion on long-term renal function in counselling.

The choice of diversion depends also on other factors besides the possible organic consequences. Bladder cancer is a complex disease because there are also social implications that involve the patient’s relationship skills and the handling of daily activities when RC is performed. Different urinary diversions may have different impacts on quality of life (QoL). This is an open topic; many aspects are involved, including, first of all, the duration of the follow-up. The perception of QoL changes with the time elapsed since the operation, and is positively or negatively influenced by the cancer outcome. Some aspects that are initially evaluated negatively over time can change and vice versa. The body integrity, the sexual sphere, and the management of the diversion are usually the factors that have the greatest impact on daily activity and, therefore, on QoL, but in a different manner between males and females. Recently, many published works have focused on QoL, but Siracusano et al. pointed out the situation in women, who are studied less with respect to the male gender, precisely highlighting the different factors, with respect to men, that impact QoL [6]. This is certainly an open topic and we do not currently have clear evidence of the superiority of one diversion over the other. Other studies are warranted.

Bladder cancer is a work in progress; after the revolution of BCG and orthotopic diversion, new horizons are now opening up in diagnosis and treatment that lead us to in-depth research.
